# Dietary supplement of Yunkang 10 green tea and treadmill exercise ameliorate high fat diet induced metabolic syndrome of C57BL/6 J mice

**DOI:** 10.1186/s12986-020-0433-9

**Published:** 2020-02-04

**Authors:** Yanzhong Zhang, Mingxing Gu, Ruru Wang, Menwan Li, Daxiang Li, Zhongwen Xie

**Affiliations:** 1 Department of Sports Sciences, Anhui Agricultural University, Hefei, Anhui People’s Republic of China; 2 State Key Laboratory of Tea Plant Biology and Utilization, School of Tea and Food Sciences and Technology, Anhui Agricultural University, 130 West Changjiang Road, Hefei, Anhui Province 230036 People’s Republic of China

**Keywords:** Yunkang 10 green tea, Exercise, Metabolic syndrome, Skeletal muscle, inflammation, RNAseq

## Abstract

**Background:**

Diet and exercise play important roles in ameliorating metabolic syndrome. Yunkang 10 (*Camellia sinensis var. assamica*) is a most cultivated tea variety for making tea in the Southwestern China. Currently, there is no report of healthy effects of Yunkang 10 green tea (YKGT) and treadmill exercise (Ex) on high fat diet induced metabolic syndrome (MetS). We aimed to investigate the beneficial effects and molecular mechanism of YKGT and Ex using high fat diet induced MetS of C57BL/6 mice.

**Methods:**

Catechins and caffeine in water extract of YKGT were measured via high performance liquid chromatography (HPLC). 10-week old mice were fed with high fat diet (HFD) for 10 weeks to induce obese mice. Then the obese mice were fed with continuous high fat diet (HFD), HFD with YKGT, HFD with Ex, and HFD with both YKGT and Ex for 8 weeks, respectively. The another group of 10-week old mice fed with low fat diet (LFD) were used as control.

**Results:**

HPLC data revealed that YKGT has abundantly high concentration of epigallocatechin gallate (EGCG) and caffeine compared to Longjing 43 (*Camellia sinensis var. sinensis*) green tea. YKGT and Ex significantly decreased the level of blood glucose, serum total cholesterol (TC), triglyceride (TG), insulin, and alanine aminotransferase activity (ALT) when compared to HFD group. The fatty liver and hepatic pro-inflammatory gene expression in the YKGT, Ex and YKGT+Ex groups was mitigated significantly compared with HFD group, respectively. The phosphorylation of inhibitor of nuclear factor kappa-B kinase α/β (IKKα/β) and inhibitor of nuclear factor kappa-B α (IkBα) protein in the nuclear factor kappa-light-chain-enhancer of activated B cells (NF-kB) signaling pathway was also decreased in YKGT or YKGT+Ex groups. The combination of YKGT and Ex prevented gene expression for lipid synthesis in the liver tissue, and significantly upregulated mRNA level of glucose transport genes in the skeletal muscles, when compared to the HFD group.

**Conclusions:**

This study demonstrated that YKGT supplement or exercise appeared to reverse preexisting metabolic syndrome, and effectively relieved the fatty liver and hepatic inflammatory response induced by high fat diet. YKGT supplement and treadmill exercise together had better beneficial effects than only one intervention.

## Introduction

Metabolic Syndrome (MetS) is a group of chronic metabolic disorders including obesity, hyperglycemia, dyslipidemia and hypertension. The risk for developing MetS is closely related to dietary and lifestyle, such as consumption of a high-fat diet, less exercise. MetS has been a strong risk factor for cardiovascular disease, diabetes and cancer [1]. Over the past decades, rapid development of economy and technology has resulted in increasing food supply and declining physical activity [2, 3]. Sedentary lifestyle and over eating habits are considered to be primarily responsible for the growing rate of obesity, diabetes and other MetS associated diseases across the world [4, 5]. The data from International Diabetes Federation (IDF) showed that the global diabetes population reaches 425 million, and undiagnosed diabetes has 212 million in 2017. According to the World Health Organization (WHO) in 2016, nearly 2 billion adults worldwide were overweight and, of these, more than half a billion were obese. Although the great progress has been made in the treatment of MetS by surgery and pharmacology, some potential side effects are inevitable all the time [6, 7]. At present, lifestyle improvement, diet intervention and exercise therapy are the basic strategies for prevention of MetS. Recent findings indicated that extended sedentary time in individuals with type 2 diabetes is associated with glucose intolerance and higher risk for development of cardiovascular diseases (CVD) [8, 9]. Aerobic exercise reduces blood pressure in both hypertensive and normotensive persons, increase insulin sensitivity and enhance immune function [10, 11]. Regular exercise training increases oxidative capacity, lipid metabolism, reduces serum triglycerides, blood pressure, insulin resistance [12, 13].

Tea has been very popular in the world. And its consumption is only second to water [14]. It has been widely recognized that tea has healthy effects on lipid lowing and anti-obesity [15, 16]. In the processing of green tea, polyphenol oxidase is inactivated by either steaming or pan-frying fresh tea leaves to save the polyphenols. Green tea contains high amount of a monomeric polyphenolic compound known as catechin and (−)-epigallocatechin gallate (EGCG), which is a major component of catechins and was the most studied in recent years. Many reports have previously demonstrated that catechins, especially EGCG, improve insulin resistance [17, 18], promote fat oxidation, lower blood total cholesterol and triglycerides, and reduce body weight [14, 19, 20]. Yunkang 10 (*Camellia sinesis var. assamica* cv. Yun Kang 10), is a widely cultivated tea cultivar in Southwestern China. It bears the advantages of wide range of adaptability, low-cost cultivation, and quick growth rate over other’s cultivars. The genome, chemical profiling, volatile components, entophytic bacteria of Yunkang 10 have been reported [21–24]. Until now, there is no report to evaluate its healthy values against MetS. Because teas are consumed by drinking water infusion or grinding powders of whole-leaf teas, it is very curious to know the beneficial effects and underlying mechanism by dietary supplement of whole tea powder using HFD induced obese mice.

Previous studies reported that a combination of green tea and exercise facilitates sports performance and endurance capacity, and effectively prevents obesity [25–27]. However, there is limited information on therapeutic effect of green tea supplement combined with aerobic exercise for ameliorating excisting MetS. The goal of this study is to investigate if the combination of Yunkang 10 green tea supplement and physical exercise has synergistically therapeutic effects on MetS induced by HFD in C57BL/6 J mice, and if so, what are underlying mechanisms?

## Materials and methods

### Samples preparation and analysis of catechins and caffeine

Tea cultivar Yunkang 10 (*Camellia sinensis var. assamica*) was cultivated in Menghai County, Yunnan Province, China. Yunkang green tea (YKGT) was provided by Tea Research Institute of the Yunnan Province. Tea cultivar Longjing 43 (*Camellia sinensis var. sinensis*) was from tea garden of Anhui Agricultural University, Anhui Province, China. Longjing green tea (LJGT) was prepared by a professional tea producer following a standard protocol. The main catechins and caffeine were analyzed following our previous protocol [14] on a Waters High Performance Liquid Chromatography (HPLC) system supported with a Waters 600 controller and Waters 2489 UV/Visible Detector (280 nm).

### Animal experiments design and treatment

Male C57BL/6 J mice (age of 9 weeks, weight 26.1 ± 0.26 g) were purchased from the National Resource Centre of Model Mice (Nanjing, China) and allowed to acclimate to the environment for 1 week on a specific pathogen free (SPF) laboratory animal facility center at Anhui Agricultural University. The animal facility was controlled with a constant temperature (22 ± 1 °C) and humidity (50 ± 5%) under a 12:12 h light–dark cycle falls on 8:00 a.m. to 8:00 p.m. All animals were housed in cages and provided with food and water ad libitum.

At 10 weeks of age, the mice were divided into low-fat-diet group (LFD, *n* = 10), and high-fat-diet group (HFD, *n* = 40). The LFD has 11% of energy derived from fat, 3.6 kcal/g (AIN93) and HFD is 60% of energy derived from fat, 5.2 kcal/g. YKGT was crushed into powder by BLENDER 800S (Warning, Corp., Torrington, CT, USA), and was added into HFD at the concentration of 5% (w/w). All diets were obtained from Trophic Animal Feed High-Tech Co., Ltd. (Nantong, China). Metabolic syndrome was induced by feeding the animals with a high fat diet for 10 weeks ad libitum. The model is consistent with previously published [28, 29].

At 20 weeks of age, 10-week HFD induced obese mice were randomly divided into four groups: HFD group (HFD, *n* = 10), HFD + YKGT group (GT, *n* = 10), HFD + Ex group (Ex, *n* = 10), and HFD + YKGT+Ex group (GT + Ex, *n* = 10). The exercise mice received a forced treadmill (Zhenghua Bio-Equipment Co, Anhui, China) running for 8 weeks (6 days per week) during the experimental period. Mice were placed on the treadmill in their respective lanes undisturbed for 15 min for their acclimated to the treadmill prior to exercise. In order to avoid injury, the mice were warmed up at slow initial speeds at 6 m per min before running at higher speed at 8 m per min. Additional file 1: Table S1 shows the detailed treadmill running schedule.

Food intake and water consumption were monitored every day. Body weight was obtained with a weight scale weekly. Fasting blood glucose was recorded using Nova StatStrip XpresstM Glucose CR Meter (Nova Biomedical, Waltham, UK) with Nova StatStrip XpresstM Glu-test Strips (Nova Biomedical, Waltham, UK) every week. All the animal experimental procedures imposed in this study were in accordance with guidelines of institutional animal care and use committee (IACUC) of Anhui Agricultural University.

### Serum and tissue samples collection

At 28 weeks of age, after 8 weeks therapeutic treatment, upon overnight fasting, animals were anesthetized by intraperitoneal injection of pentobarbital sodium (50 mg/kg), and were sacrificed after peripheral blood collection from the ophthalmic vein. Serum was obtained by centrifugation at 3000 rpm/min for 5 min at 4 °C, then stored at − 80 °C. Liver weight was measured on a scale. All other tissue samples were harvested for further biochemical, molecular and immunostaining analyses. A small piece of liver tissues and a piece of soleus muscle tissues were preserved in RNA stabilization solution (Thermo Fisher Scientific, Baltics, USA) for gene expression analysis and RNAseq, respectively. And small pieces of liver tissues were fixed in formaldehyde solution (ZHANYUN, Wuxi, China) for histological experiment, and the rest of liver tissues were immediately liquid nitrogen frozen before stored at − 80 °C for protein expression studies.

### Serum biochemical analysis

The levels of triglyceride (TG), low-density lipoprotein cholesterol (LDL), high-density lipoprotein cholesterol (HDL), and total cholesterol (TC) in the serum were measured using micro test kits (Johnson Medical Equipment Co., Ltd., Shanghai, China). Serum insulin level was measured using the protocol of Rat/Mouse Insulin ELISA Kit (EMD Millipore Corporation, Billerica, MA, USA). The enzymatic activity of alanine transaminase (ALT) was analyzed with enzyme kits in accordance with the manufacturer’s instruction (Nanjing, Jiancheng Biotechnology Co., Ltd., Nanjing, China). OD values were determined by enzyme micro-plate reader (SpectraMaxR i3X Molecular Devices, LLC. California, USA).

### Quantitative real-time PCR

Total RNA was extracted from liver tissue fixed in RNA stabilization solution using Trizol reagent (Vazyme Biotech Co., Ltd., Nanjing, China) following the manufacturer’s instruction. The 2 μg of total RNA was reverse transcribed to cDNA using Hiscript II 1st strand cDNA Synthesis Kit (Vazyme Biotech Co., Ltd., Nanjing, China). Real-Time PCR was performed with SYBR Green Master Mix using Real-Time PCR Detection System (CFX96 Touch, Bio-Rad, USA) following the previous protocol [30], the expression of 36B4 was used as housekeeping gene to normalize target gene [31]. Primer sequences are designed for mice and listed in Additional file 2: Table S2.

### Western blot analysis

Western blot was performed following the method described previously [32]. In brief, a small piece of liver tissue was immersed in 10% trichloroacetic acid solution and frozen at − 80 °C for 24 h. The 100% acetone was used to wash the liver sample to remove trichloroacetic acid. Then the liver tissue was homogenized in 2XSDS buffer. The protein concentration was determined by BCA Assay kit (Vazyme Biotech Co., Ltd., Nanjing, China). The equal amount of total proteins were separated by SDS polyacrylamide gel electrophoresis and transferred to blotting membranes. The membranes were blocked with defatted-milk buffer, and were further incubated at 4 °C overnight with primary antibodies including total-IKKβ, phosphorylation-IKKα/β, total-IκBα, phosphorylation-IκBα, total and phospho-Akt (Ser473), total and phospho-PI3 Kinase p85 (Tyr458)/p55 (Tyr199), and total and phospho-mTOR (Ser2448) (Cell Signaling Technology, MA, USA), fatty acid synthase (FAS), sterol regulatory element binding protein-1 (SREBP1) and β-actin (Santa Cruz, CA, USA). The membranes were incubated with appropriate HRP conjugated secondary antibody (Santa Cruz, CA, USA). Protein bands were visualized using ECL developing solution (Vazyme Biotech Co., Ltd., Nanjing, China). The intensities of protein expression were analyzed using Image J software. β-actin was used as a internal control.

### Hematoxylin-eosin staining

Briefly, the fixed liver tissues were dehydrated and embedded in paraffin (Paraplast Tissue Embedding Medium, LEICA, USA) using modular tissue embedding system (LEICA EG1150 H, USA). The 5 μm sections were cut by using fully automated rotary microtome (LEICA RM2255, USA) and mounted in positive charged slides (Adhesion Microscope Slides, CITOGLAS, China). Hematoxylin-eosin (HE) staining was carried out using HE staining kit (Boster Biological Technology Company, China). All the images were acquired by microscope (LEICA DM500, USA) at 200X. Hepatic adipose infiltration cells were counted manually using Image J software.

### RNA sequencing and data analysis

In each group, three soleus skeletal muscle samples were randomly selected to sequence the transcripts. The TruSeq library was prepared following the method previously described [33]. The fragments per kilobase of transcript per million fragments mapped (FPKM) were calculated by dividing the read count of each transcriptional model with its length and scaling the total per sample to a million, and were used to indicate the expression levels of each sample. A gene expression value in FPKM equal or greater than 1 was considered to be expressed in the sample. The raw sequence data was analyzed following our published method [33]. Briefly, DESeq R package (1.10.1) was applied to analyze differential expression of two groups. The Benjamini and Hochberg approach was used to adjust the resulting *p* values for controlling the false discovery rate. Genes with a DESeq adjusted *p* value of < 0.05 were assigned as differentially expressed.

### Statistical analysis

All results were presented as mean ± S.E.M. Graph Pad Prism5 software was used for statistical analysis. Multiple groups were compared by one-way or two-way ANOVA with Tukey^’^s test when appropriate. Student’s t-test was conducted to determine significantly difference between specific two groups. Differences show statistically significant when *P* < 0.05.

## Results

### The contents of catechins and caffeine in Yunkang 10 green tea

Green teas are rich in catechins, which were proved to have beneficial effects. Yunkang 10 is a “big leaves” tea plant (*Camellia sinesis var. assamica* cv. Yunkang 10), and Longjing 43 is a “small leaves” tea plant (*Camellia sinesis var. sinensis* cv. Longjing 43), which is one of the most cultured tea cultivars in Eastern China. Here, we comparatively measured the contents of catechins and caffeine in Yunkang 10 green tea (YKGT) and Longjing 43 green tea (LJGT). Our data showed that YKGT has obviously higher concentration of total catechins and caffeine compared to LJGT. Among catechins, the amounts of EGCG and epicatechin gallate (ECG) are higher, however epigallocatechin (EGC), + catechin (+C), gallocatechin gallate (GCG) are lower in YKGT than that of LJGT (Table 1).

**Table 1:**
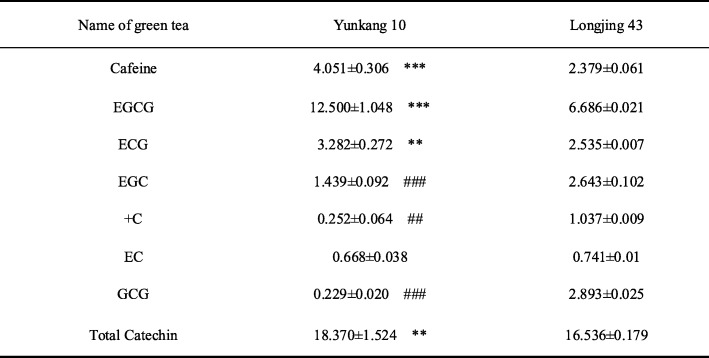
Relative concentration of catechin and caffeine in Yunkang 10 and Longjing 43 green tea (Dry weight %)

Data were expressed as mean±SEM (*n*=3). ** *p* < 0.01, *** *p* < 0.001 when compared with Longjing 43 up-regulation

^##^ *p* < 0.01, ^###^ *p* < 0.001 when compared with Longjing 43 down-regulation

### YKGT and Ex ameliorated phenotypes of MetS in HFD induced C57BL/6 J mice

**Fig. 1:**
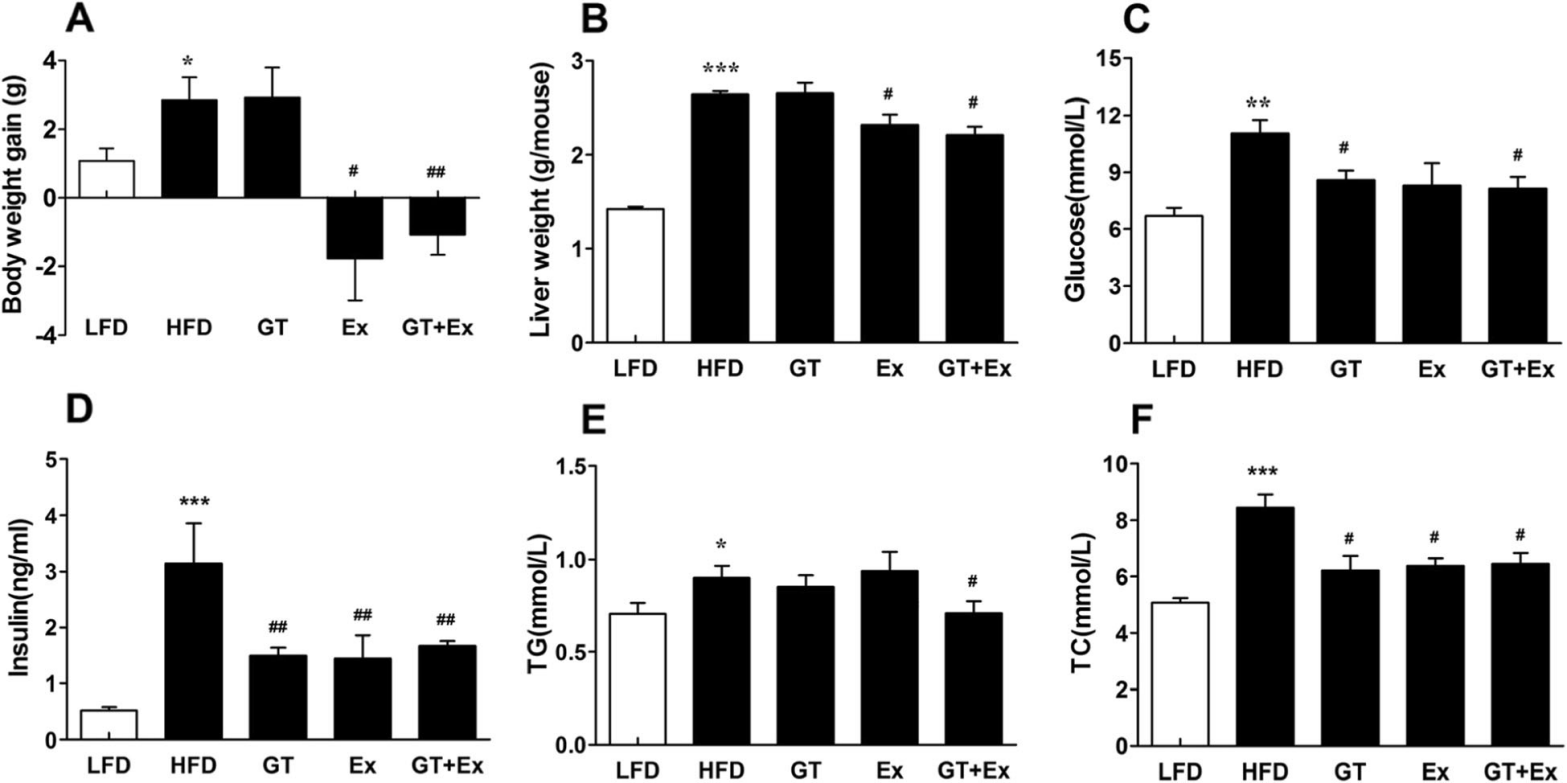
Improvement of phenotypes of MetS of HFD mice treated with GT, Ex or GT + Ex for 8 weeks. Notes: Body weight gain (**a**); liver weight (**b**); serum glucose (**c**); insulin (**d**), total cholesterol, TC (**e**), triglyceride, TG (**f**). Values given are mean ± SEM, *n* = 5~7.; *P < 0.05, **P < 0.01, ***P < 0.001 when compared with LFD; ^#^*P* < 0.05, ^##^*P* < 0.01 when compared with HFD

MetS characterized by obesity, diabetes and dyslipidemia. HFD induced obese mice significantly increased body weight and liver weight (Fig. 1a-b), observed higher concentration of serum glucose, insulin, TC and TG (Fig. 1c-f) compared to LFD mice. The treatment of obese mice with YKGT for 8 weeks significantly decreased the elevated glucose, insulin and TC level in plasma (Fig. 1c, d, f), but did not have obvious effects on reducing body weight, liver weight and plasma TG level (Fig. 1a, b, e). Ex for 8 weeks reduced body weight, liver weight, as well as decreased plasma insulin and TG level of obese mice. However, treatment of obese mice with combination of YKGT and Ex significantly deceased body weight, liver weight, and attenuated the elevation of all observed plasma parameters, glucose (by 26.3%), insulin (by 46.8%), TG (by 21.3%) and TC (by 23.7%) compared to continuous HFD feeding obese mice, respectively (Fig. 1c-e).

**Fig. 2:**
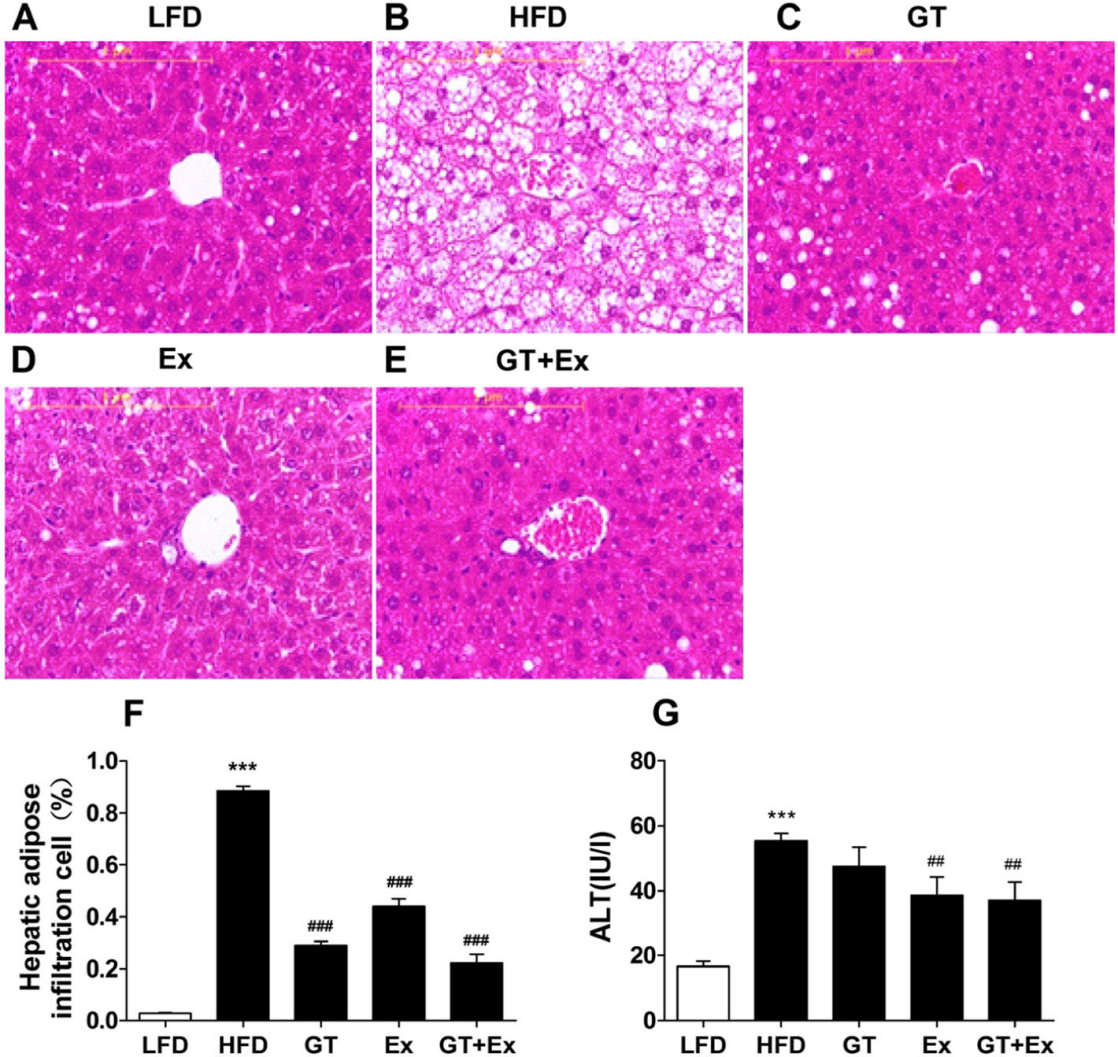
YKGT, Ex and YKGT+EX diets reverse hepatic steatosis and reduce ALT activity. Notes: Hematoxylin–eosin HE staining showing the liver of LFD feeding mice (**a**); HFD feeding mice (**b**); HFD with YKGT supplement feeding mice (**c**); HFD feeding with exercise mice (**d**); HFD with YKGT supplement feeding and exercise group mice (**e**); scale bars, 1 μm. Semi-quantification of hepatocytes with adipose (**f**); serum ALT activity (**g**). *N* = 5~7. ***P < 0.001 when compared to LFD; ^##^*P* < 0.01, ^###^*P* < 0.001 when compared to HFD

Histopathological sections of liver tissue illustrated that the HFD mice displayed aberrantly fatty hepatocytes. In contrast, clear hepatic lobule without any lipid droplets of normal hepatic architecture was observed in LFD mice. However, treatment with YKGT, Ex or both YKGT and Ex restored the normal liver architecture with minimal deposition of fat droplets in hepatocytes (Fig. 2a-f). In addition, treatment of obese mice with Ex or YKGT plus Ex significantly decreased the activity of alanine aminotransferase (ALT) in liver of obese C57BL/6 mice (Fig. 2g).

### YKGT and Ex improved liver lipid and glucose metabolism in HFD induced C57BL/6 J mice

**Fig. 3:**
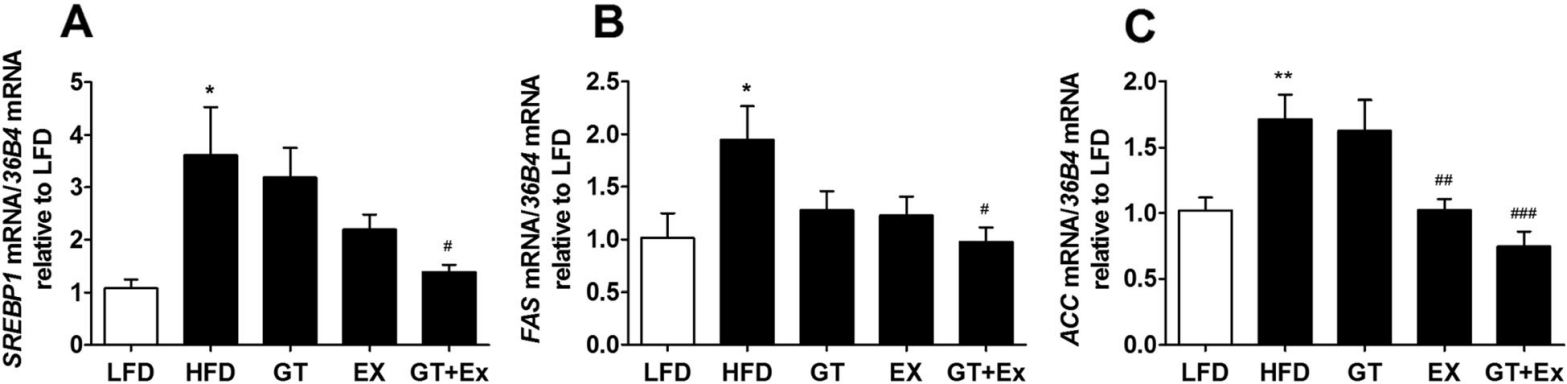
YKGT+Ex suppress HFD induced increase of the mRNA expression of genes involved in lipid synthesis in the mice liver. Notes:LFD, LFD feeding mice; HFD, HFD feeding mice; GT, HFD with YKGT supplement feeding mice; Ex, HFD feeding with exercise mice; GT + Ex, HFD with YKGT supplement feeding and exercise group mice. *SREBP1* (**a**), *FAS* (**b**), *ACC* (**c**), Values given are mean ± SEM, *n* = 5~7. *P < 0.05, **P < 0.01 when compared with LFD; ^#^*P* < 0.05, ^##^*P* < 0.01, ^###^*P* < 0.001 when compared with HFD

**Fig. 4:**
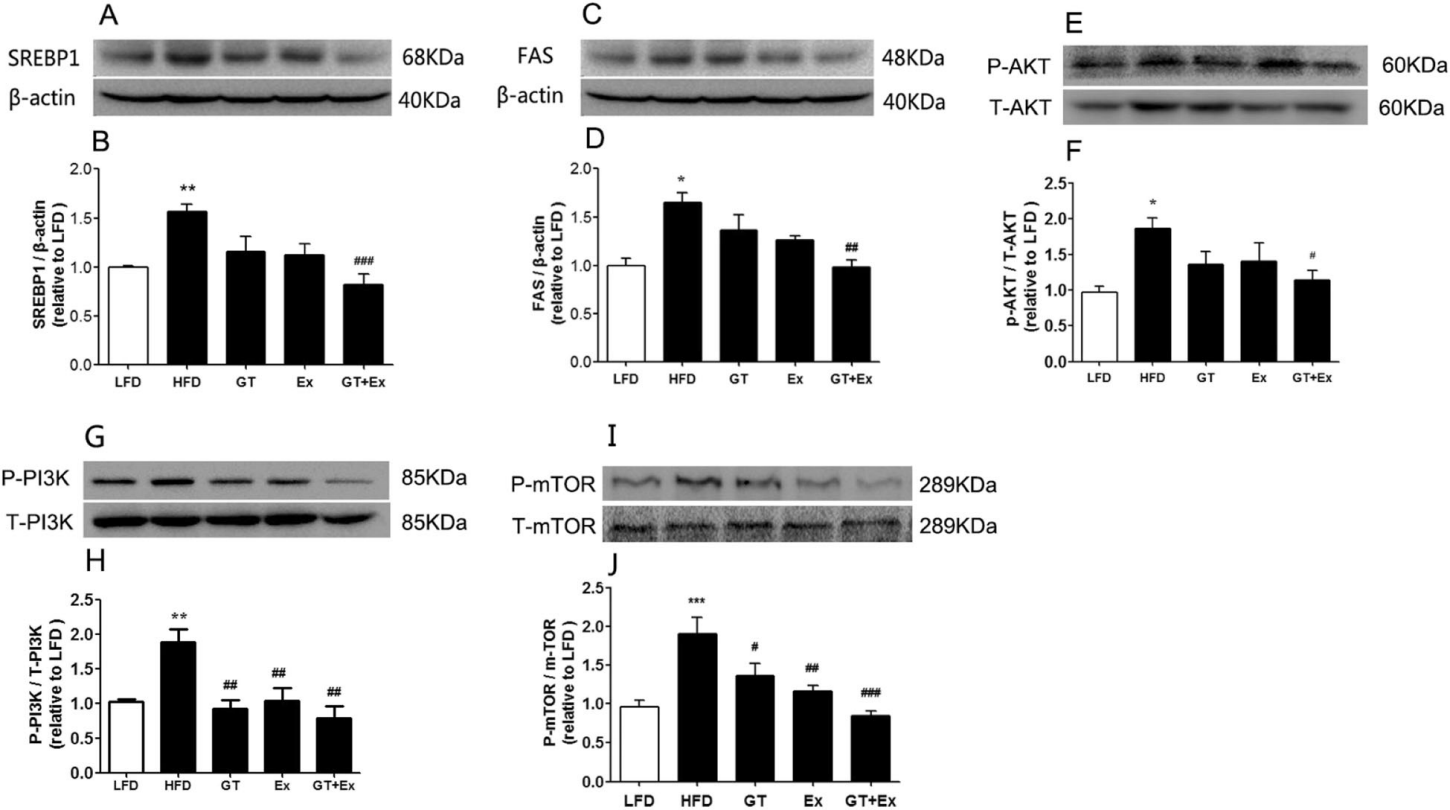
YKGT+Ex attenuate HFD induced increase of the lipid synthesis and glycometabolism related protein expression in the mice liver. Notes: LFD, LFD feeding mice; HFD, HFD feeding mice; GT, HFD with YKGT supplement feeding mice; EX, HFD feeding with exercise mice; GT + Ex, HFD with YKGT supplement feeding and exercise group mice. Representative image (**a**) and summary data (**b**) for SREBP1 protein (*n* = 4); Representative Fig. (**c**) and summary data (**d**) for FAS protein (*n* = 4); Representative image (**e**) and summary data (**f**) for Total and P-AKT protein (*n* = 4); Representative Fig. (**g**) and summary data (**h**) for total and P-PI3K protein (*n* = 4); Representative Fig. (**i**) and summary data (**j**) for total and P-mTOR protein (*n* = 4). Values are mean ± SEM, *P < 0.05, **P < 0.01,***P < 0.001 when compared to LFD; ^#^*P* < 0.05, ^##^*P* < 0.01, ^###^*P* < 0.001 when compared with HFD

High fat diet significantly up-regulated the expressions of fatty acid synthesis gene *Srebf1, Fas,* and *Acc* in the liver of HFD group mice compared to LFD group mice (Fig. 3a-c). Dietary supplement with YKGT alone for 8 weeks did not prevent the overexpression of all these three fatty acid synthesis genes. And Ex alone only showed significant down-regulation *ACC* gene expression (Fig. 3c). However, the expression level of *SREBF1, FAS,* and *ACC* all was decreased significantly by combination of YKGT and Ex intervention (Fig. 3a-c). This observation was further demonstrated by the results of western blotting. The hepatic protein expression of both SREBF1 and FAS decreased dramatically in the combined intervention group mice compared to HFD mice (Fig. 4a-d).

Akt (Protein Kinase B)- PI3K (phosphatidylinositol 3-kinase) and mTOR (The mammalian target of rapamycin) signaling pathway plays a pivotal role in regulating metabolism of lipid and glucose. Our data showed that phosphorylation of PI3K-Akt-mTOR were all elevated in the liver of HFD group mice. YKGT or Ex intervention decreased the phosphorylation of PI3K and mTOR, respectively. However, YKGT plus Ex attenuated the phosphorylation of all three proteins in the liver of treated group mice (Fig. 4e-j).

### YKGT and Ex ameliorated liver inflammation in HFD induced C57BL/6 J mice

**Fig. 5:**
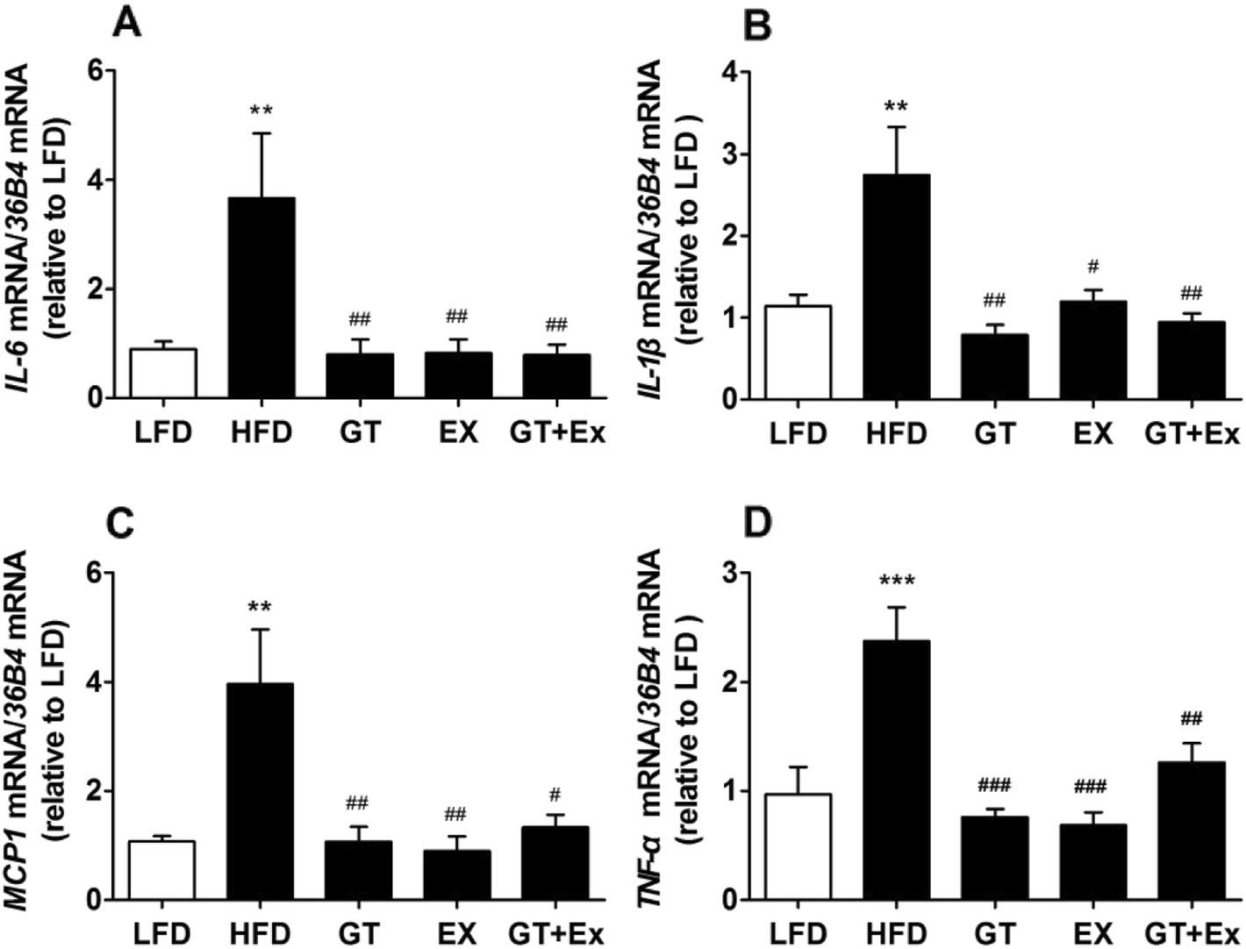
GT, EX and GT + Ex suppress the HFD induced up-regulation of inflammatory cytokines gene expression level in the liver of various group mice. Notes: LFD, LFD feeding mice; HFD, HFD feeding mice; GT, HFD with YKGT supplement feeding mice; EX, HFD feeding with exercise mice; GT + Ex, HFD with YKGT supplement feeding and exercise group mice. *IL-6* (**a**), *IL-1β* (**b**), *MCP1* (**c**), *TNF-α* (**d**). Values are mean ± SEM, *n* = 5~7. **P < 0.01 and ***P < 0.001 when compared to LFD; ^#^*P* < 0.05, ^##^*P* < 0.01, ^###^*P* < 0.001 when compared to HFD

Chronic inflammation is a main pathological cue for MetS. Liver inflammation can cause fatty liver disease. The mRNA expression profile of pro-inflammatory cytokines of *IL-6*, *IL-1β*, *MCP-1* and *TNF-α* was observed in liver tissue. Our Real Time PCR data showed that expression of all these pro-inflammatory cytokines was significantly up-regulated in the liver of HFD group mice than that of mice fed with LFD. However, treatment of HFD mice with YKGT, Ex or both YKGT and Ex for 8 weeks resulted in profound down-regulation of the cytokines genes expression in mice liver tissue (Fig. 5a-d).

**Fig. 6:**
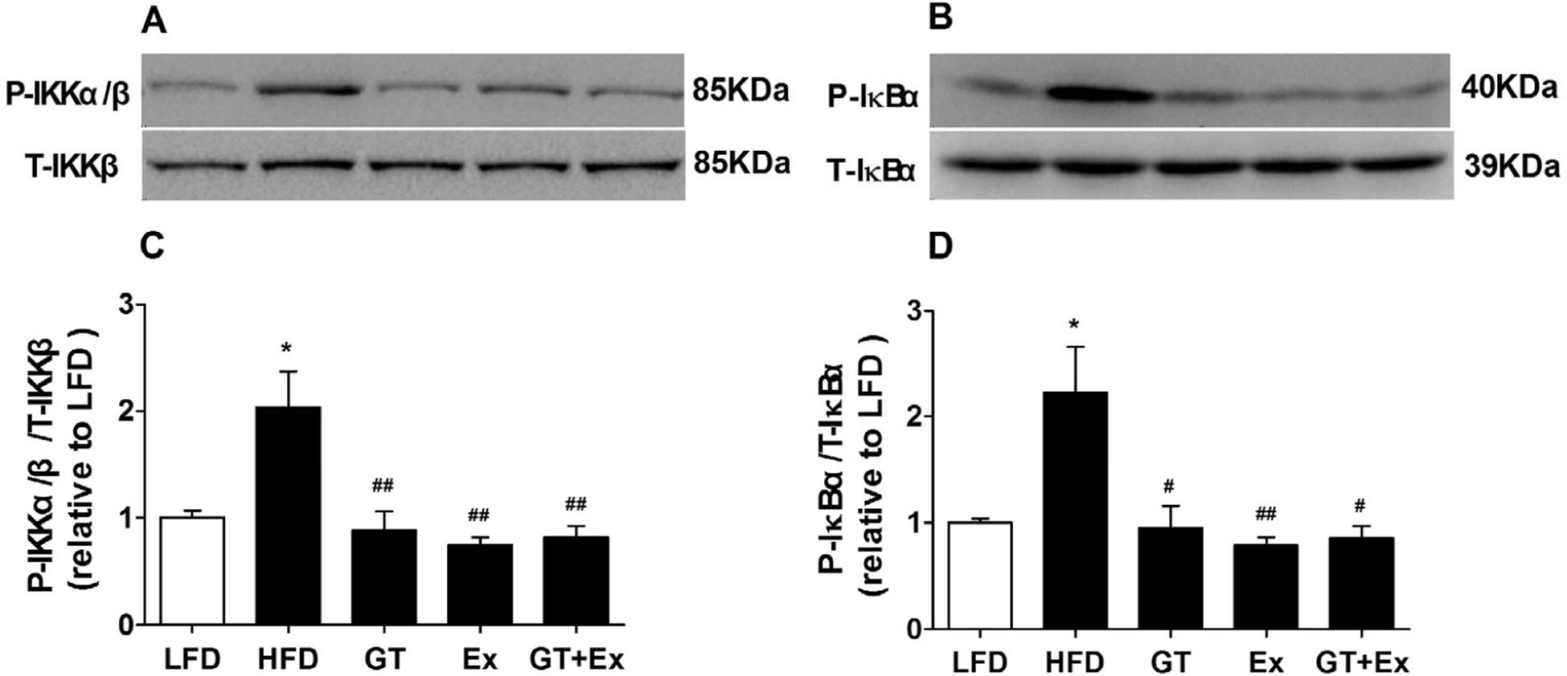
GT, EX and GT + Ex attenuate HFD induced increase of phosphorylated IKKα/β and IκBα protein in the mice liver. Notes: LFD, LFD feeding mice; HFD, HFD feeding mice; GT, HFD with YKGT supplement feeding mice; EX, HFD feeding with exercise mice; GT + Ex, HFD with YKGT supplement feeding and exercise group mice. Representative image (**a**) and summary data (**c**) of P-IKKα/β (*n* = 5~7), and representative image (**b**) and summary data (**d**) of P- IκBα (*n* = 5~7). Values are mean ± SEM,*P < 0.05 when compared to LFD, ^#^*P* < 0.05, ^##^*P* < 0.01 when compared to HFD

Nuclear factor kappa light chain enhancer of activated B cells (NF-κB) is a family of highly conserved transcription factors and a key regulator for cellular inflammatory response. The phosphorylation of inhibitor of κB kinases (IKK) and inhibitor of κBα (IκBα) is a key process for activating inflammatory response of NF-κB pathway. Western blotting data showed that YKGT, Ex or YKGT+Ex therapy dramatically prevented the increase of phosphorylated protein expression of IKKα/β and IκBα in the liver compared to HFD mice (Fig. 6 a-d).

### YKGT and Ex altered protein and lipid metabolism and inflammatory pathways in skeletal muscle of HFD induced C57BL/6 J mice revealed by RNAseq data

**Fig. 7:**
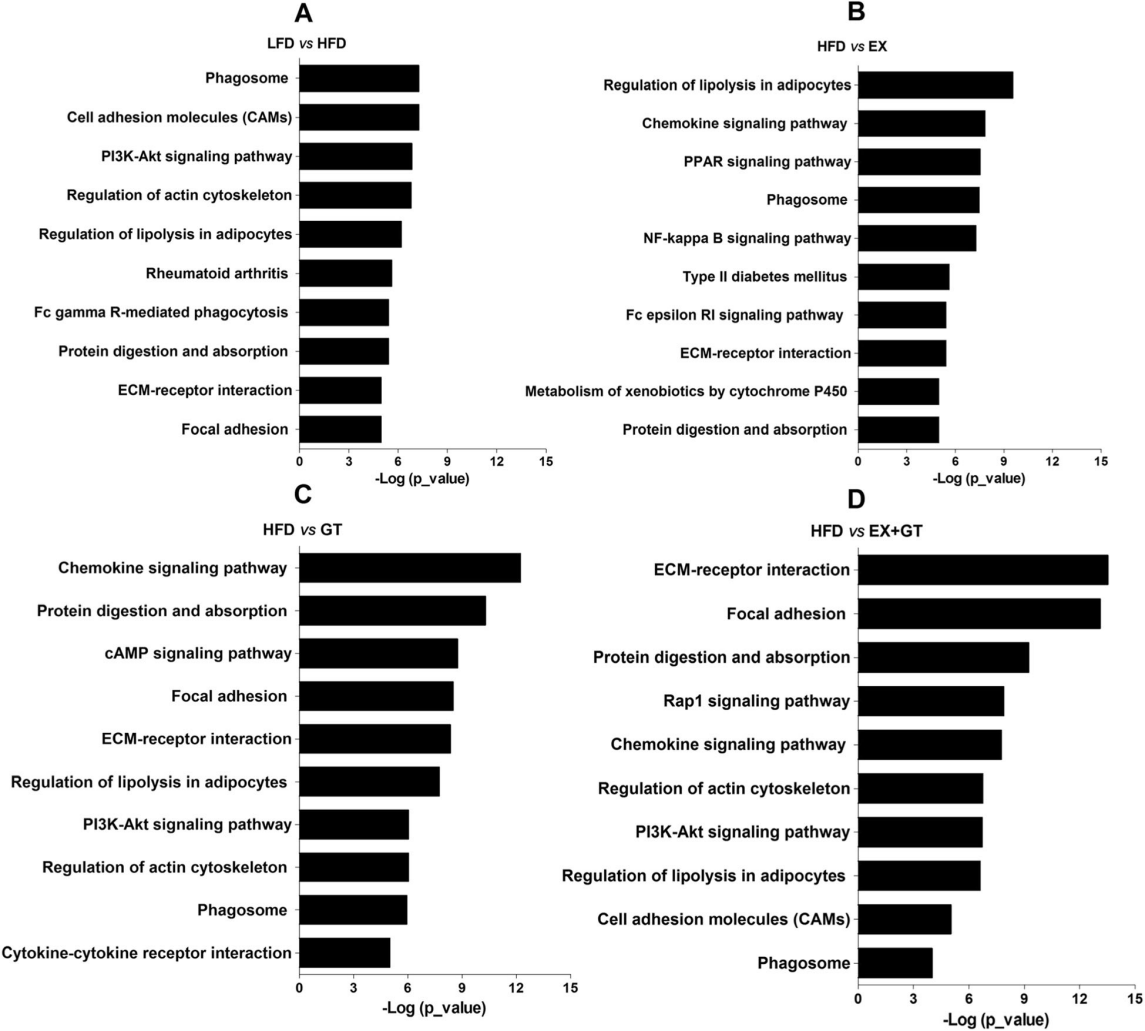
Overview of KEGG annotation of all DEGs identified by the RNAseq analysis on mice muscle tissues treated with GT, Ex or GT + Ex for 8 weeks. Notes: The top 10 KEGG terms identified in the comparisons are reported. X axis represents the *p*-values in log 10 scale. Y axis represents functional classification of KEGG. LFD mice versus HFD mice (**a**), HFD mice versus HFD mice plus exercise (**b**), HFD mice versus HFD mice feeding with HFD supplement with YKGT (**c**), and HFD mice versus HFD mice feeding with HFD supplement with YKGT and exercise (**d**)

Liver and muscle are two important tissues for nutrients metabolism. High-throughput sequencing technology was used to analyze RNAseq data from skeletal muscle tissues. Transcriptome sequencing results showed that 1946 genes were up-regulated in the skeletal muscle of HFD group mice compared to that of the LFD group mice. In addition, the numbers of up-regulated genes were significantly reduced after treatment with YKGT, Ex and YKGT plus Ex. (Additional file 3: Figure S1). KEGG pathway analysis of the differentially expressed genes (DEGs) from LF VS HF showed that phagosome and nutrients metabolic pathways (regulation of lipolysis and protein digestion and absorption) are most altered pathways; HF VS Ex DEGs analysis revealed that nutrients metabolic pathways (regulation of lipolysis, PPAR signaling pathway, and protein digestion and absorption) and inflammatory pathway (phagosome, chemokine signaling pathway, NF-κB signaling pathway) are most pathways involved; HF VS YKGT DEGs analysis indicated that inflammatory pathway (chemokine signaling pathway, cytokine-cytokine receptor interaction and phagosome) and nutrients metabolic pathways (protein digestion and absorption, regulation of lipolysis, and PI3K-AKT signaling pathway) are most involved; and HF VS YKGT+Ex DEGs analysis illustrated that ECM receptor interaction, skeletal muscle cell focal adhesion, and nutrients metabolic pathways (protein digestion and absorption, regulation of lipolysis), as well as inflammatory pathway (chemokine signaling pathway, and phagosome) are mostly modulated in skeletal muscle (Fig. 7 a-d).

### YKGT and Ex ameliorated the expression of genes involved in inflammation and glucose metabolism in skeletal muscle of HFD induced C57BL/6 J mice

**Fig. 8:**
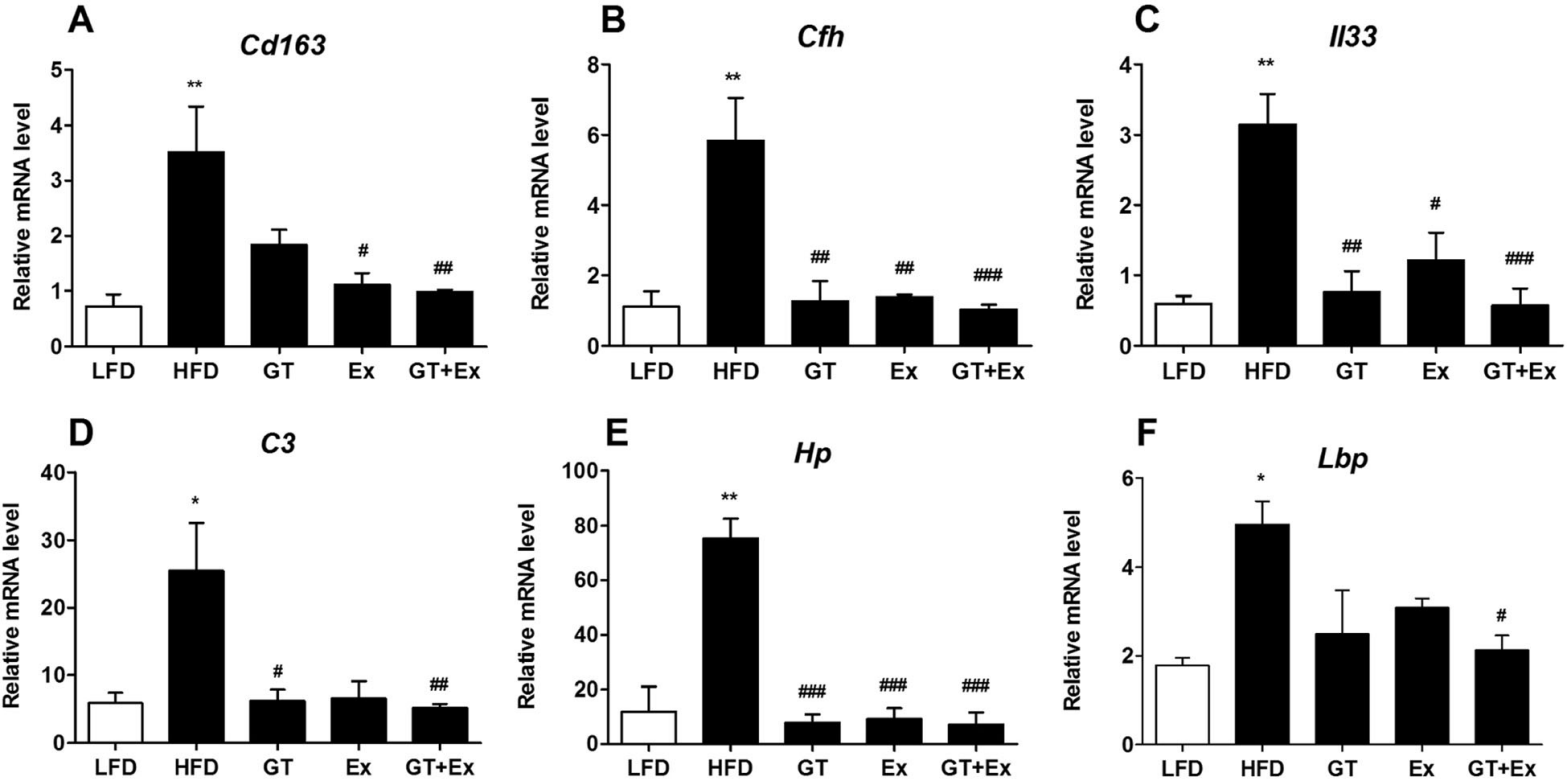
The inflammatory response gene expression was quantified by FPKM in the muscle tissue of various group mice. Notes: LFD, LFD feeding mice; HFD, HFD feeding mice; GT, HFD with YKGT supplement feeding mice; EX, HFD feeding with exercise mice; GT + Ex, HFD with YKGT supplement feeding and exercise group mice. The mRNA levels of Cd163 (**a**)*, Cfh* (**b**)*, IL33* (**c**)*, C3* (**d**)*, Hp* (**e**), *Lbp* (**f**) are showing. Values are means ± SE, *n* = 3. *P < 0.05, **P < 0.01 when compared to LFD, ^#^*P* < 0.05, ^##^*P* < 0.01, ^###^*P* < 0.001 compared to HFD

**Fig. 9:**
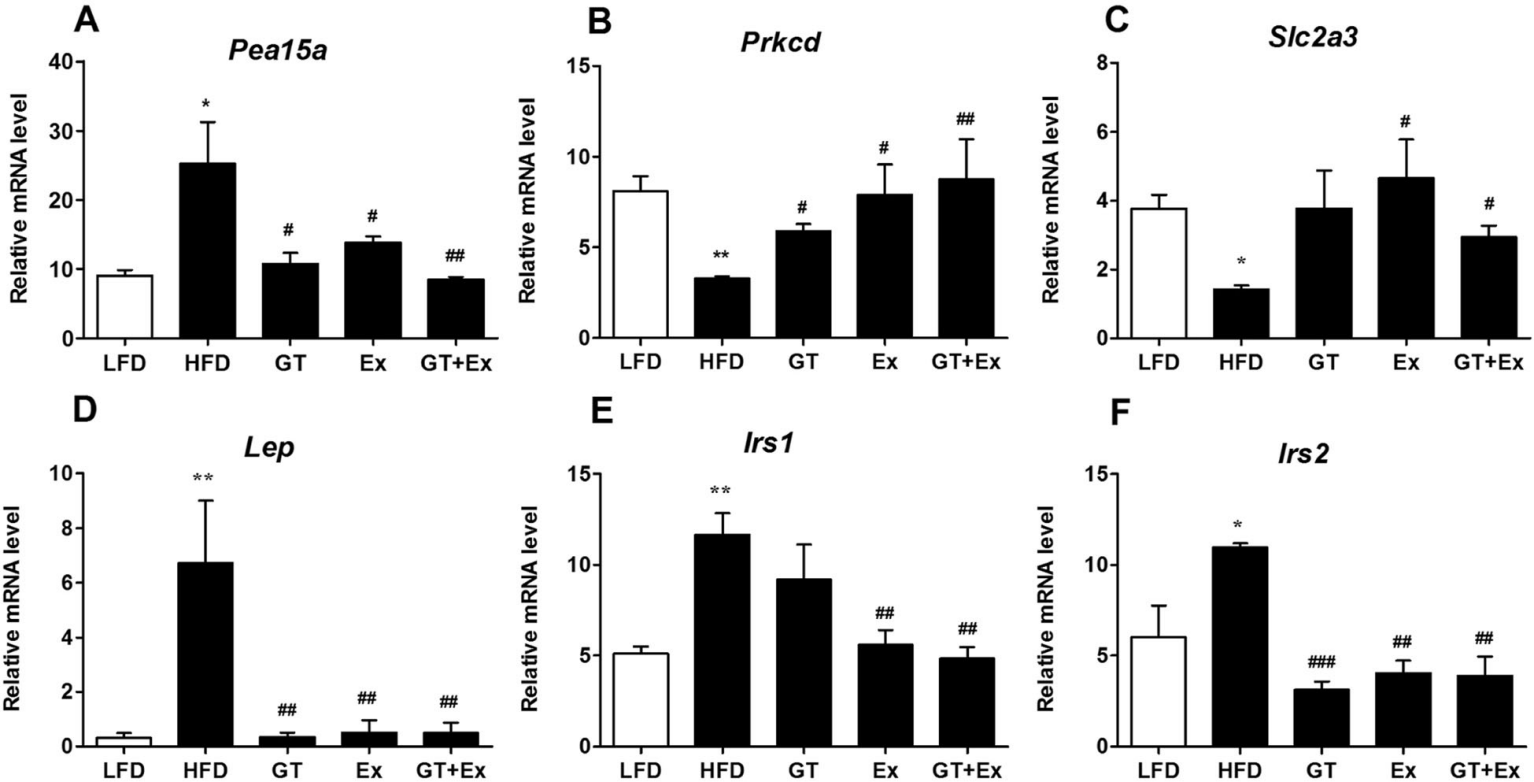
The glucose transport related gene expression was quantified by FPKM in the muscle tissue of various group mice. Notes: LFD, LFD feeding mice; HFD, HFD feeding mice; GT, HFD with YKGT supplement feeding mice; EX, HFD feeding with exercise mice; GT + Ex, HFD with YKGT supplement feeding and exercise group mice. The mRNA levels of *Pea15a* (**a**)*, Prkcd* (**b**)*, Slc2a3* (**c**)*, Lep* (**d**), *Irs1* (**e**), and *Irs2* (**f**) are showing. Values are means ± SE (*n* = 3). *P < 0.05, **P < 0.01 compared with LFD, ^#^*P* < 0.05, ^##^*P* < 0.01, ^###^*P* < 0.001 when compared with HFD

Previous analysis of RNAseq data showed that the DEGs were mainly involved in inflammation and nutrients metabolism response to YKGT, Ex or YKGT plus Ex intervention. Based on the gene quantification in Fragments Per Kilobase of transcript per Million mapped reads (FPKM), we found that inflammatory related genes *Cd163, Cfh, Il33, C3, Hp, Lbp* were down-regulated in the skeletal muscle of YKGT, Ex or YKGT plus Ex group mice compared to HFD induced mice (Fig. 8a-f). And YKGT plus Ex treatment had better effects than that of YKGT or Ex alone. Glucose transport related genes *Prkcd,* and *Slc2a3* were significantly increased by YKGT, Ex or YKGT plus Ex intervention compared to HFD mice (Fig. 9 b, c), *Pea15a*, *Lep, Irs1 and Irs2* were obviously decreased in the skeletal muscle by YKGT, Ex or YKGT plus Ex treatment (Fig. 9a, d-f).

## Discussion

Yunkang 10 has been widely cultivated in Southwestern China. However, the potential health effects and relevant molecular mechanisms of YKGT are still unknown. Preventive effects of MetS by green tea and catechins, especially EGCG have been extensively investigated [14–19, 34]. Until today, there is only limited information on therapeutic effects of green tea or EGCG intervention on ameliorating existed MetS [35–37]. During recent years, several studies have reported that a combination of green tea and exercise facilitates sports performance and endurance capacity, and effectively prevents obesity [25–27, 38, 39]. However, the underlying mechanisms are still unclear. The present study investigated therapeutic effects and molecular mechanisms of the combination of YKGT supplement and physical exercise using 10 week HFD induced MetS mice model, which mostly represented MetS of human population.

Obesity, hyperlipidemia and insulin resistance are the major features of the MetS. The mice fed with HFD for10 weeks showed typical MetS phenotype, which include obese, hyperglycemia, hyperlipidemia and hyperinsulinemia, as well as fatty liver. The HFD mice were then treated with YKGT, Ex or YKGT plus Ex for additional 8 weeks. The results showed that treatment with YKGT alone for 8 weeks did not prevent the body weight and liver weight gain, and triglyceride (TG) increase (Fig. 1 a, b, e), but did ameliorate the increase of serum glucose, insulin and total cholesterol level (Fig. 1c, d, f), compared to continuous HFD feeding group mice. Ex alone did restrain the body weight and liver weight gain (Fig. 1a, b), prevented the increase of insulin and total cholesterol level (Fig. 1d, f), and did not inhibit the increase of plasma TG level compared to continuous HFD feeding group mice. However, YKGT plus Ex prevented all these index increases. A randomized trial reported the effect of green tea supplement and interval sprinting exercise (ISE) on the body composition of overweight males, and found that ingestion of green tea by itself did not result in a significant decrease in body or abdominal fat, but increased fat utilization during submaximal exercise. And the combination of 12 weeks of GT ingestion and ISE did not result in greater total and abdominal fat reduction compared to 12 weeks of ISE alone [26]. This result indicated that GT ingestion might not contribute to fat reduction in overweight males. This finding is close correlated to our data that YKGT supplement did not prevent the body weight and liver weight gain, and TG increase. Another randomized control trial suggested that GT catechin consumption enhances exercise-induced deduction in abdominal fat and serum TG [27]. Martin et al. reported that short-term GT supplementation did not affect glucose kinetics following ingestion of an oral glucose load post exercise. However, GT was associated with attenuated insulinemia [40]. Recently, Amozadeh et al. reported a randomized trial for overweight and obese females who participating aerobic training (AT) and green tea supplementation on cardio metabolic risk factors, and found that GT plus AT had better effects on decreasing body weight, body fat percentage, body mass index (BMI), TG, LDL, blood pressure, and heart rate (HR) than that of GT or AT alone [41]. These results are consistent with our finding.

Next, we investigated the molecular mechanism underlying YKGT and Ex prevented MetS induced by HFD. Sterol regulatory element-binding protein 1 (*SREBP1*), Fatty acid synthase (*FAS*) and Acetyl-CoA carboxylase (*ACC*) are critical enzymes for fatty acid synthesis. Our data showed that the either mRNA expression of *SREBP1*, *FAS*, or protein expression of SREBP1, FAS were altered by intervention of YKGT or Ex alone in the liver of mice. However, treatment of mice with YKGT plus Ex significantly prevented the increase of gene expression of *Srebp1*, *Fas* and *ACC* (Fig. 3a-c), as well as protein expression of SREBP1 and FAS (Fig. 4a-d). Previous studies reported that GT ameliorated hyperglycemia and improved blood lipid parameters by regulating the expression of *FAS, ACC, SREBP-1* genes [42–44]. PI3K-Akt-mTOR signaling pathway was activated in the liver of HFD mice, and YKGT and Ex intervention block this activation by phosphorylation (Fig. 4e-j). PI3K-Akt signaling pathway modulated lipid synthesis genes by regulating transcriptional factor l SREBP-1 [45]. In addition, PI3K-Akt-mTOR1 participated lipid and glucose metabolism by regulating the secretion of very-low-density lipoprotein (VLDL) cholesterol, oxidation of fatty acids and hepatic gluconeogenesis [46]. Tea intake prevented lipid accumulation in the liver and adipose tissues [47, 48]. Exercise can effectively increase fat oxidation and energy consumption [49, 50]. Previous studies reported that GT supplement combined with exercise enhanced fat oxidation in HFD mice and obese adults [39, 51]. In addition to promote energy expenditure, this study demonstrated that YKGT plus Ex also effectively suppressed fatty acid synthesis in the liver of HFD mice.

Inflammatory response plays a momentous role in the development of MetS [52, 53]. Numerous inflammatory cytokines are involved in the process of MetS developing into diabetes and cardiovascular disease [54]. Our results showed that YKGT, Ex and YKGT plus Ex significantly prevented the over-expression of pro-inflammatory cytokines *IL-6, TNF-α, IL-1β* and *MCP1* induced by HFD in the mice liver (Fig. 5a-d). And RNAseq data from skeleton muscle tissue also showed that inflammatory related genes, *Cd163, Cfh, Il33, C3, Hp, Lbp* were all down-regulated by YKGT, Ex or YKGT plus Ex treatment (Fig. 8a-f). Ma S et al. found that Ketogenic Diet and exercise significantly decreased IL-6 concentration in gastrocnemius and plasma [55]. Recently, Cialdella-Kam et al. reported that four-week supplementation of quercetin or green tea extract (GTE) or quercetin plus GTE did not altered inflammatory cytokine level of IL-1β, IL-6, TNF-αand IFN-γin the plasma of 12-week HFD mice [56]. The difference between our data and this report might due to the different intervention period (8 weeks vs. 4 weeks).

Proinflammatory cytokines are directly regulated by the activation of NFκB. The activation of NF-κB pathway is a central part of the complex network of inflammatory response. The NF-κB proteins contain multiple subunits, p50 and p65 dimer bounds to IκBα localized in the cytosol under unstimulated condition. The inhibitor of κB kinase (IKK) complex is phosphorylated once stimulated, and then activated IKK phosphorylated IκBα proteins, which releases p50 and p65 dimer from complex, p65 transfers to cell nucleus, where binds to specific sites of DNA in promoter region, and induce the synthesis of pro-inflammatory cytokines in the cells [57]. Therefore, the phosphorylation of IKK and IκBα is a key event for NFκB activation. In this study, YKGT, Ex and YKGT plus Ex all blocked the phosphorylation of IKKα/β and IκBα, and thus inhibited the transcript of pro-inflammatory cytokines. GT polyphenol, major EGCG has found to have an anti-inflammation function. Ueno T et al. found that EGCG decreased the lipid droplets in hepatic cells and prevented liver injury by the inhibition of NF-kB pathway [42]. In addition, Tipoe et al. illustrated that EGCG decreased the DNA-binding activity of NF-kB and the expression of TNF-α, which ameliorated liver inflammation and fibrosis in carbon tetrachloride (CCl_4_)-induced liver injury in mice [58].

Besides generating force for movement, skeletal muscle also contributes to health through the use and storage of macronutrients. RNAseq data from soleus muscle tissues showed that treatment with YKGT, Ex and YKGT plus Ex significantly down-regulated most of genes induced by HFD (Additional file 3: Figure S1). KEGG pathway analysis of DEGs revealed that nutrients metabolic pathways (regulation of lipolysis, PPAR signaling pathway, and protein digestion and absorption) and inflammatory pathways (chemokine signaling pathway, NF-κB signaling pathway and phagosome) are most modulated pathways response to YKGT, Ex or YKGT plus Ex intervention. Hodgson et al. suggested that long term GTE intake accelerates fat metabolism at rest, while facilitates the expression of fat metabolism enzyme genes in the skeletal muscles during exercise, thus decreases adipogenic genes in the liver [19]. Previous studies also demonstrated that a combination of tea catechin intake and frequent exercise prevents obesity efficiently by accelerating fat oxidation in the liver and skeletal muscles and facilitates energy expenditure [27, 59]. Recently, a report found that theabrownin and swinging exercise prevented obesity and insulin resistance by accelerating metabolism of glucose and lipid [60]. Emerging data have revealed that obesity may also negatively alter muscle protein turnover, or the breaking down and rebuilding of functional proteins [61]. Feeding and resistance exercise reduced stimulation of myofibrillar protein synthesis in obese people [62]. One paper reported that GT catechins suppress muscle inflammation and hasten performance recovery after exercise [63].

Type 2 diabetes results from defects in glucose transport in skeletal muscle. And the glucose transport gene 4 (GLU4) is abundant present in the skeleton muscles [64]. Tsai et al. reported that 8-week oral GTE supplementation increased post exercise systemic fat oxidation and exercise-induced muscle GLUT4 protein content [65]. From RNAseq data, we found that glucose metabolic related genes *Prkcd* and *Slc2a3* were promoted by YKGT, Ex or YKGT plus Ex. PRKCD is activated by diacylglycerol (DAG) and involves in glucose metabolic pathway. SLC2A3 is a glucose transmembrane transporter, and facilitates glucose transport in the muscle cell [66]. Obesity decreased sensitivity to leptin, developed leptin resistance [67]. Our RNAseq data showed that YKGT, Ex or YKGT plus Ex significantly decreased leptin gene expression in the skeletal muscle. Murase et al. reported that combination of tea-catechin intake and regular swimming significantly decreased serum leptin level in diet induced obesity C57BL/6 mice [68].

## Conclusion

**Fig. 10:**
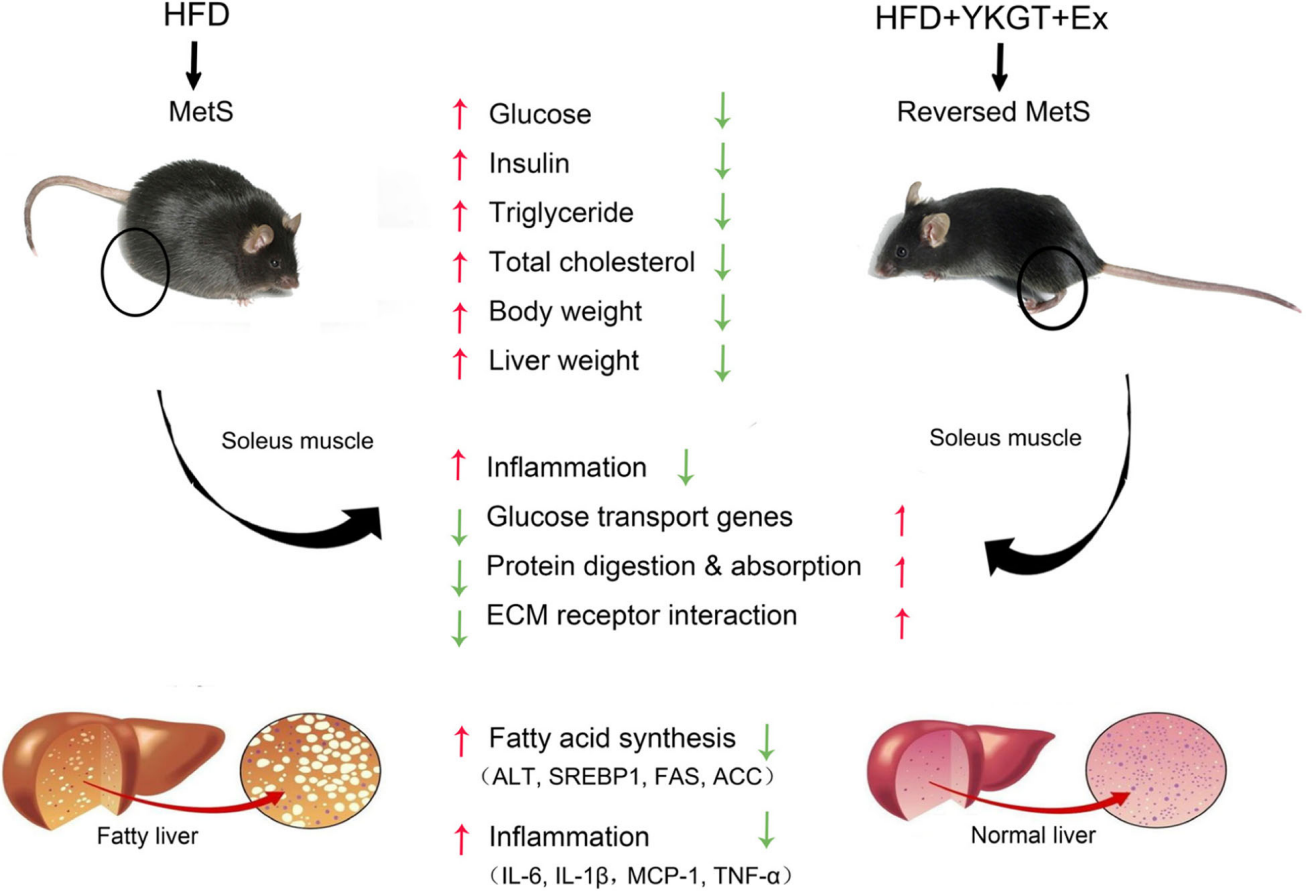
The schematic diagram of YKGT+Ex reversed HFD induced MetS of C57BL/6 J mice. Note: HFD, HFD feeding mice; GT, HFD + GT + Ex, HFD with YKGT supplement feeding and exercise group mice. MetS, Metabolic Syndrome;  ALT, Alanine transaminase; SREBP1, Sterol regulatory element-binding transcription factor 1;  FAS’ Fatty acid synthase; ACC, Acetyl-CoA carboxylase; IL-6, Interleukin 6; IL-1β, Interleukin 1β; MCP-1, monocyte chemoattractant protein 1; TNF-α, tumor necrosis factor-α

YKGT is characterized by high concentration of total catechins and caffeine. YKGT supplement only significantly prevented the increase of plasma glucose, insulin and TC level, and Ex alone ameliorated body weight and liver weight increase, and decreased insulin and TC level. However, YKGT plus Ex prevented all these parameters increase, and had better therapeutic effects than that of YKGT or Ex alone for HFD induced MetS mice. HE staining showed that YKGT plus Ex reversed fatty liver, and decreased the serum ALT activity. Mechanistic studies revealed that combination of YKGT and Ex significantly suppressed the key lipid synthesis genes and protein expression in the liver, and significantly upregulated glucose transport genes expression in the skeletal muscles, when compared to the HFD group mice. In addition, the hepatic pro-inflammatory gene expression was mitigated significantly by inhibition of NF-kB activation through decreasing the phosphorylation of IKKα/β and IkBα by YKGT, Ex and YKGT plus Ex intervention. RNAseq data from soleus muscle tissues showed that treatment with YKGT, Ex and YKGT plus Ex significantly down-regulated most of genes induced by HFD. KEGG pathway analysis indicated that nutrients metabolic pathways and inflammatory pathways are the most modulated pathways response to YKGT, Ex or YKGT plus Ex intervention compared to HFD mice. The schematic diagram of YKGT plus Ex reversed HFD induced MetS of C57BL/6 J mice is showing in Fig. 10.

This study demonstrated that combination of YKGT supplement and aerobic exercise appeared to reverse preexisting MetS, and effectively relieved the fatty liver and hepatic inflammatory response induced by HFD. YKGT supplement and aerobic exercise together might be a beneficial strategy for ameliorating MetS of human population. However, adequate intensity and appropriate period of exercise intervention, and YKGT dosage for treatment of MetS require further investigation.

## Supplementary information

**Additional file 1: Table S1.** The treadmill exercise schedule for HFD mice during the experiment.

**Additional file 2: Table S2.** Primer sequences used for RT-PCR experiment.

**Additional file 3: Figure S1.** Diagram representing the percent of genes differentially expressed in each comparison.

## Data Availability

The datasets used and/or analysed during the current study are available from the corresponding author on reasonable request.
