# Supplementary material for: Dietary supplement of Yunkang 10 green tea and treadmill exercise ameliorate high fat diet induced metabolic syndrome of C57BL/6 J mice
Source: Nutr Metab (Lond). 2020 Feb 4;17:14. doi: 10.1186/s12986-020-0433-9 (PMC7001212; doi:10.1186/s12986-020-0433-9)
Supplement: Supplementary file 1 — Additional file 1: Table S1. The treadmill exercise schedule for HFD mice during the experiment. [file 12986_2020_433_MOESM1_ESM.docx]

Table S1: The treadmill exercise schedule for HFD mice during the experiment
