# Supplementary material for: Dietary supplement of Yunkang 10 green tea and treadmill exercise ameliorate high fat diet induced metabolic syndrome of C57BL/6 J mice
Source: Nutr Metab (Lond). 2020 Feb 4;17:14. doi: 10.1186/s12986-020-0433-9 (PMC7001212; doi:10.1186/s12986-020-0433-9)
Supplement: Supplementary file 2 — Additional file 2: Table S2. Primer sequences used for RT-PCR experiment. [file 12986_2020_433_MOESM2_ESM.docx]

# Table S2: Primer sequences used for RT-PCR experiment.
