# Supplementary material for: Dietary supplement of Yunkang 10 green tea and treadmill exercise ameliorate high fat diet induced metabolic syndrome of C57BL/6 J mice
Source: Nutr Metab (Lond). 2020 Feb 4;17:14. doi: 10.1186/s12986-020-0433-9 (PMC7001212; doi:10.1186/s12986-020-0433-9)
Supplement: Supplementary file 3 — Additional file 3: Figure S1. Diagram representing the percent of genes differentially expressed in each comparison. [file 12986_2020_433_MOESM3_ESM.docx]

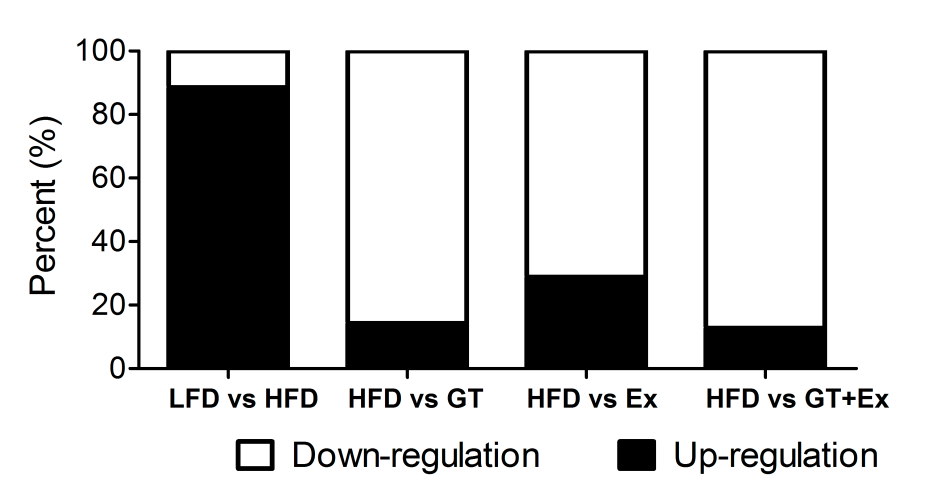


**Figure S1.** Diagram represents the percent of genes differentially expressed in muscle tissue of each comparison.

Notes: DEGs were classified as Up-regulated or Down-regulated (DEGs with FDR < 0.05, |Fold change| > 1 were included).
